# Production of Omegas-6 and 9 from the Hydrolysis of Açaí and Buriti Oils by Lipase Immobilized on a Hydrophobic Support

**DOI:** 10.3390/molecules23113015

**Published:** 2018-11-18

**Authors:** Malena Martínez Pérez, Enrico Cerioni Spiropulos Gonçalves, Jose Carlos Santos Salgado, Mariana de Souza Rocha, Paula Zaghetto de Almeida, Ana Claudia Vici, Juliana da Conceição Infante, Jose Manuel Guisán, Javier Rocha-Martin, Benevides Costa Pessela, Maria de Lourdes Teixeira de Moraes Polizeli

**Affiliations:** 1 Departamento de Bioquímica e Imunologia, Faculdade de Medicina de Ribeirão Preto, Universidade de São Paulo, Ribeirão Preto, São Paulo 14049-900, Brazil; malenac@usp.br (M.M.P.); spiropulos.enrico@gmail.com (E.C.S.G.); mariana_rocha@usp.br (M.d.S.R.); paulazag@usp.br (P.Z.d.A.); julianainfante@usp.br (J.d.C.I.); 2 Departamento de Química, Faculdade de Filosofia, Ciências e Letras de Ribeirão Preto, Universidade de São Paulo, Ribeirão Preto, São Paulo 14049-901, Brazil; salgado@usp.br; 3 Departamento de Biologia, Faculdade de Filosofia, Ciências e Letras de Ribeirão Preto, Universidade de São Paulo, Ribeirão Preto, São Paulo 14040-901, Brazil; acvici@usp.br; 4 Departamento de Biocatálisis, Instituto de Catálisis y Petroleoquímica, CSIC, Campus UAM, Cantoblanco, 28049 Madrid, Spain; jmguisan@icp.csic.es (J.M.G.); javirocha@icp.csic.es (J.R.-M.); 5 Departamento de Biotecnología y Microbiologia de los Alimentos, Instituto de Ciencias de la Alimentación, CIAL-CSIC, Campus UAM, Cantoblanco, 28049, Spain: b.pessela@csic.es; 6 Departamento de Engenharia e Tecnologias, DET- Instituto Superior Politecnico de Tecnologias e Ciências-ISPTEC, Av. Luanda Sul, Rua Lateral Via S10, Talatona-Republica de Angola

**Keywords:** lipase, *Beauveria bassiana*, *Fusarium oxysporum*, immobilization, octyl-sepharose, omega-6, omega-9, Açaí (*Euterpe oleracea* Martius), Buriti (*Mauritia flexuosa*)

## Abstract

This paper describes a bioprocess to obtain omegas-6 and 9 from the hydrolysis of Açaí (*Euterpe oleracea* Martius) and Buriti *(Mauritia flexuosa)* oils by lipases immobilized on octyl-sepharose. For this, oils and butters were initially selected as the carbon source which resulted in higher production of lipases in *Beauveria bassiana* and *Fusarium oxysporum* cultures. The carbon source that provided secretion of lipase by *B. bassiana* was Açaí oil, and for *F. oxysporum*, Bacuri butter. Lipases obtained under these conditions were immobilized on octyl-sepharose, and both, the derivatives and the crude extracts were biochemically characterized. It was observed that the immobilization promoted an increase of stability in *B. bassiana* and *F. oxysporum* lipase activities at the given temperatures and pH. In addition, the immobilization promoted hyperactivation of *B. bassiana* and *F. oxysporum* lipase activities being 23.5 and 11.0 higher than free enzyme, respectively. The hydrolysis of Açaí and Buriti oils by the derivatives was done in a biphasic (organic/aqueous) system, and the products were quantified in RP-HPLC. The results showed the potential of these immobilized lipases to obtain omegas-6 and 9 from Brazilian natural oils. This work may improve the enzymatic methodologies for obtaining foods and drugs enriched with fatty acids.

## 1. Introduction

Nowadays, the production of omega fatty acids, as oleic acid (omega-9) and linoleic acid (omega-6), from several vegetable oils, is of great relevance to the oleo-chemical, food and pharmaceutical industries [1,2]. Omega-6 and 9 fatty acids are important components present in the diet. Among omega-6 rich foods we can mention sunflower, corn, cottonseed, soybeans and sesame oils. In the case of omega-9, it should be noted, canola, soybean, avocado, sunflower and almond butter [2,3].

Linoleic acid (omega-6), unlike oleic (omega-9), is considered an essential fatty acid, since it is not produced by the body metabolism and must be acquired through the diet or supplements [4]. The balance of these acids in the organism is related to good health, and the lack of this balance can lead to the development of numerous diseases. Omegas have several roles in the metabolism and contribute to the proper functioning of the human organism.

Linoleic acid (omega-6) is related to the formation of arachidonic acid (AA), an important precursor of eicosanoids, which are small molecules that act as hormones in inflammatory processes and they are also important components of the immune system [5]. Additionally, linoleic acid (omega-6) helps to reduce nerve pain in people with diabetic neuropathy and favors the formation of anti-inflammatory precursors. These components act reducing the inflammation observed in cancer, diabetes, arthritis, Alzheimer’s disease, and heart diseases [6]. Linoleic acid (omega-6) also helps in hair growth, in the symptoms of Attention Deficit Hyperactivity Disorder, and reduces blood pressure [6,7]. 

Oleic acid (omega-9) improves the level of HDL-cholesterol (“good cholesterol”) and decreases the amount of LDL-cholesterol (“bad cholesterol”) in the blood, consequently, reduces the risk of developing cardiovascular diseases and strokes [8,9]. In addition, oleic acid (omega-9) consumption is associated with a better physical performance, increased energy availability, and good mood [10]. Oleic acid (omega-9) has shown to be a promising therapeutic agent for the treatment of cognitive deficits associated to pathologies like Alzheimer’s disease, promoting memory improvement [11].

Brazilian Amazon Forest is a great source of a wide variety of oils and butters extracted from fruits with excellent nutritional properties. The oils of Açaí *(Euterpe oleracea* Martius) and Buriti (*Mauritia flexuosa*) are good examples of Amazon oils; they are rich in monounsaturated and polyunsaturated fatty acids. They are used in the food, pharmaceutical, and cosmetic industries [12,13,14]. Buriti oil has approximately 73.3–78.73% of oleic acid (omega-9) and 2.4–3.93% of linoleic acid (omega-6), while Açaí oil is composed of 60% of oleic acid (omega-9) and 12% of linoleic acid (omega-6) [15,16,17]. The high amount of omega fatty acids present in the composition of these oils makes them excellent raw materials for obtaining omega fatty acids.

Recently, the use of enzymes to obtain mono- and polyunsaturated fatty acids by the industry has shown advantages over traditional chemical methods: chemical reactions under milder conditions; easy separation of products from the reaction mixture when immobilized; lower costs and the absence of undesirable products [18,19]. Lipases (triacylglycerol hydrolases EC 3.1.1.3) are hydrolases that catalyze the hydrolysis of triacyl glycerides, releasing glycerol and free fatty acids [20]. These enzymes have a catalytic mechanism characteristic based on conformational changes, called interfacial activation, allowing the hydrolysis of insoluble oil molecules [21]. These enzymes are produced by several plants, microorganisms, and mammals. Today, numerous fungal strains that can produce lipases are being studied and identified by various industries [22]. Microbial lipases are the most used group of enzymes for biotechnological applications and organic chemistry [20]. However, to our knowledge, the use of lipases obtained from strains of *B. bassiana* and *F. oxysporum* for the hydrolysis of Açai and Buriti oils, aiming to obtain oleic (omega-9) and linoleic acids (omega-6) have not yet been described in the literature. Under the experimental conditions presented in this work, the study of biochemical characteristics and hydrolysis efficiency of lipases produced by these fungi became important.

The industrial processes in which lipases participate usually require enzyme stabilization, biocatalyst reuse, and their use for an extended period, allowing greater economy and efficiency. A simple tool that is very useful for improving the properties of the enzymes is the immobilization in hydrophobic supports [23,24]. This immobilization strategy involves the surroundings of the lipase active site, allowing the maintenance of an open conformation, which in many situations can cause its hyperactivation and stabilization. In addition, enzymatic immobilization on hydrophobic surfaces is a reversible process, which allows lipase desorption from the support, facilitating the recovery and reuse of the enzyme in new processes [25,26,27]. Other properties as purity, selectivity or specificity can also be optimized through immobilization [28,29,30]. However, immobilization on hydrophobic supports has some drawbacks, such as the release of the enzyme to the reaction medium when subjected to higher temperatures or higher concentrations of organic co-solvents [31]. In addition, many substrates and products of lipases have typical properties of detergents, such as long chain fatty acids, mono and diglycerides, dibutyrin and diacetin favoring enzyme desorption [32]. In order to solve these problems, the use of heterofunctional supports has been proposed [33,34]. Although such supports improve stability and prevent enzyme desorption, immobilization strategies involving covalent bonds between the enzyme and the support becomes an irreversible process, which prevents the reuse of the support. Additionally unsuccessful ligations of the enzyme with the support can lead to the decrease of activity and even enzymatic leakage [35,36]. Recently the use of polymers such as polyethyleneimine (PEI) has been described, this molecule is capable to produce reversible physical intramolecular crosslinks, forming large enzymatic aggregates, thus avoiding the release of the enzymes from the support [37,38,39,40]. In addition, PEI creates an environment that helps reduce enzyme contact with solvents, preventing protein denaturation [41]. Moreover, such PEI coating decrease the possibility of oxygen molecules to promote the oxidation of the hydrolysis products, extending the half-life of these molecules to the step of the enzymatic reaction [42,43]. Finally, this polymer contributes to the stabilization of the open conformation of the lipase lid, influencing the enantioselectivity of these enzymes [44].

Although several studies have shown the ability of lipases to hydrolyze fish oil and produce omega-3 fatty acids (EPA and DHA), [18,45,46] the use of lipases to produce omega-6 and 9 through the hydrolysis reaction of exotic oils from Brazilian Amazon has been little explored. The aim of this work was to characterize the production of linoleic acid (omegas-6) and oleic acid (omega-9) through the hydrolysis of Açaí and Buriti oils by fungal lipases, produced by *F. oxysporum* (Fo) and *B. bassiana* (Bb) immobilized on a hydrophobic support.

## 2. Results and Discussion

### 2.1. Selection of Carbon Source for Lipase Production

**Figure 1:**
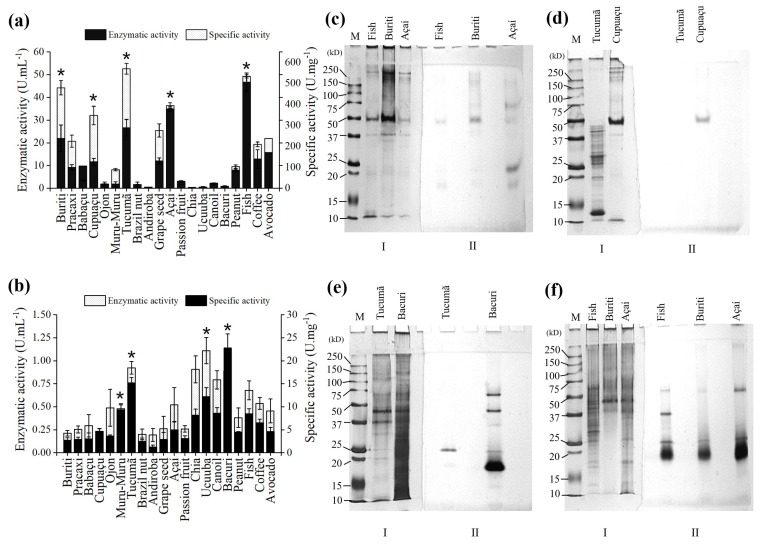
Selection of carbon source for lipase production: Total enzyme activity and specific enzyme activity of culture supernatants from *B. bassiana* (**a**) and *F. oxysporum* (**b**) with different oils and butters. The symbol (⋆) above the vertical bar indicates the difference in significance in relation to other oils, for the studied parameters of interest. The statistics used was One-Way ANOVA followed by Tukey post-hoc with ⋆ *p* < 0.05. The values are expressed in mean ± SEM, representing *n* = 3 for each oil. Polyacrylamide electrophoresis (12%) was under semi-denaturing conditions of the supernatant cultures that showed the highest enzymatic activities. (**c**) and (**d**) protein electrophoresis (**I**) and zymogram (**II**) of *B. bassiana* supernatant cultures with Tucumã, Cupuaçu, Fish, Buriti and Açaí. (**e**) and (**f**) protein electrophoresis (**I**) and zymogram (**II**) of the supernatants of *F. oxysporum* cultures with Tucumã, Bacuri, Fish, Buriti and Açaí. Lane 1: molecular mass. Other specifications are described in Section 3.1 and Section 3.2.

In the search for suitable carbon sources to induce lipase activity, 20 different oils and butters were tested as inducers of lipase production for *F. oxysporum and B. bassiana* in Adams medium. The enzyme activity was evaluated by the increase in the values of absorbance at 405 nm in 5 min, due to the hydrolysis of *p*-nitrophenyl palmitate (*p*NPP) by lipase. The best oils and butters for *B. bassiana* lipase production were: Cupuaçu (10 U·mL^−1^); Buriti (20 U·mL^−1^); Tucumã 28 U·mL^−1^); Açaí (35 U·mL^−1^) and Fish (45 U·mL^−1^) (Figure 1a). The best results for *F. oxysporum* were obtained with the following oils and butters: Muru-Muru (0.50 U·mL^−1^); Ucuuba (0.60 U·mL^−1^); Tucumã (0.75 U·mL^−1^); and Bacuri (1.10 U·mL^−1^) (Figure 1b).

Later on, the five oils and butters that showed higher lipase production were chosen to test if these carbon sources had the ability to induce more than one type of lipase; for this purpose, protein and zymogram gels were performed. The carbon source that induced a greater number of lipases in *B. bassiana* was Açaí (Figure 1c). Despite the specific activity of 50 U·mg^−1^ obtained with Tucumã butter (Figure 1a), it was not possible to detect a spot of lipase activity on the zymogram (Figure 1d). This fact could be explained by the aggressive conditions in which the SDS-PAGE run is proceeded (alkaline pH, high voltage, SDS concentration) and the distinct substrate used in the zymogram compared to the activity assay. Among all the oils and butters selected to perform the *F. oxysporum* zymogram, Bacuri butter showed the potential to induce a larger number of lipases (Figure 1e) when compared to the other oils and butters evaluated (Figure 1e and Figure 1f). This fact corroborates to the high specific activity observed (Figure 1b). The different profiles of *F. oxysporum* and *B. bassiana* lipase production observed are related to the different chemical composition of each inducing oil. Several chemical components present in these oils and butters can promote patterns of activation, which may alter the sequences in the fungi genome responsible for the generation of lipases with distinct characteristics. Another interesting aspect is that in both, Açaí oil and Bacuri butter, oleic acid is found in higher proportions [16,47,48]. This acid has been described as one of the main components that induce lipase production in fungus such as *Candida rugosa* [49], a fact that would explain the high total activities observed for lipase in the fungi studied.

### 2.2. Immobilization of B. bassiana and F. oxysporum Lipase on Octyl-Sepharose

**Figure 2:**
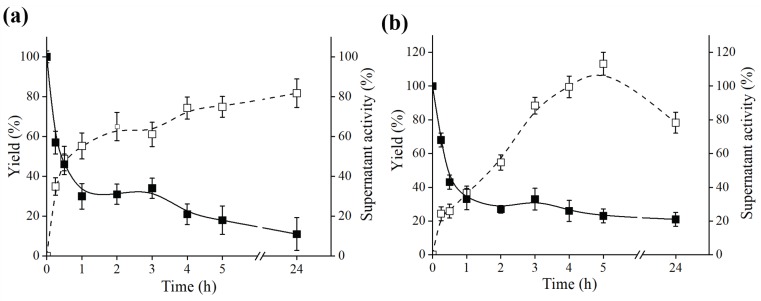
Kinetics immobilization on octyl-sepharose of *B. bassiana* and *F. oxysporum* culture supernatants: Yield increase of octyl-sepharose and decrease in the relative activity of the supernatant, expressed as percentage, as a function of the adsorption time, for (**a**) *B. bassiana* and (**b**) *F. oxysporum.* Aiming to achieve immobilization, 2 g of octyl-sepharose were added to 40 U of crude extract in sodium phosphate buffer 50 mM, pH 7.0, at 4 °C. The 100% of relative activity was considered as 40 U offered to immobilization. Activity was followed by 0.003% *p*-nitrophenyl-palmitate (*p*NPP) in McIlvaine buffer. (□) Yield (%) and (■) Supernatant activity (%). The values are expressed in means ± SEM, with *n* = 3. The SEM is lesser than 1% of the mean value (error bars are not evident, as they lie within the area of the symbol).

It was observed that at the end of the immobilization process the amount of activity adsorbed on the octyl support was 81.78% for *B. bassiana* and 78.27% for *F. oxysporum*, which proved the efficiency of immobilization on supports with hydrophobic characteristics. An interesting fact to be highlighted is that a considerable hyperactivation of lipase activity was obtained in the derivatives obtained from both fungi (Table 1 and Figure 2). For *B. bassiana*, an improvement of the lipase activity was obtained in relation to the initial activity (before the immobilization on octyl) approximately 23.5 fold, whereas for *F. oxysporum* it was 11.0 fold in relation to the initial activity. Probably, the immobilization of the lipases to the octyl support occurred by the region of the hydrophobic lid of these enzymes, allowing the exposure of the active site, thereby improving substrate accommodation. In addition, there are reports showing that the immobilization of lipases on octyl-sepharose supports increases the activity of these enzymes, as occurred with the ones isolated from microorganisms such as *Rhizopus niveus, Mucor javanicus, Pseudomonas fluorescens, Rhizomucor miehei, Humicola lanuginosa,* and *Candida antarctica;* furthermore the fact has been explained that the lipase was in its open and dissociated form [23,50,51].

Lipase activity was measured with 0.003% *p*-nitrophenyl palmitate (*p*NPP) in McIlvaine buffer pH 6.0, and the activity of soluble lipase offered for immobilization (40 U) was regarded as 100%. *Yield from supernatant* corresponds to the fraction of activity retained from supernatant to the octyl support, *relative activity of octyl derivate* represents the enzymatic activity of the derivative offered for immobilization. *Octyl derivative specific activity* corresponds to the total lipase activity present in the derivative per gram of support after immobilization (2 g). The values are expressed in means ± SEM, with *n* = 3.

### 2.3. Effect of Octyl Immobilization on B. bassiana Lipase Stability

**Figure 3:**
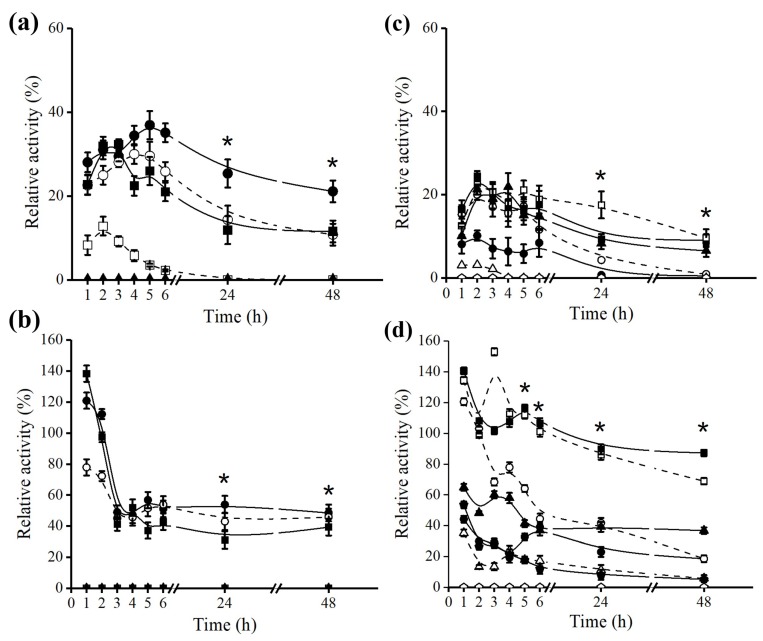
Thermal and pH stabilities of *B. bassiana* lipase: Thermal stability of crude extract (**a**) and derivative (**b**). pH stabilities of crude extract (**c**) and derivative (**d**). Thermal inactivation was performed in McIlvaine buffer pH 7.0 at the following temperatures; (●) 40 °C; (○) 50 °C; (■) 60 °C; (□) 70 °C and (▲) 80 °C. The pH stability was performed at 40 °C with McIlvaine buffer (pH 3–8) and 200 mM glycine buffer (pH 9 and 10); (●) pH 3.0; (○) pH 4.0; (■) pH 5.0; (□) pH 6.0; (▲) pH 7.0; (△) pH 8.0; (♦) pH 9.0 and (◊) pH 10.0. The lipase activity was obtained by 0.003% *p*NPP assay and relative activity was expressed according to the initial activity of the derivative prior to incubation at different pH and temperatures at several time intervals. The 100% of relative activity was considered as 940 U of *B. bassiana* lipase activity immobilized on octyl measured at the beginning of the experiment. Other specifications are described in Section 3.8 and Section 3.9. The symbol (⋆) above an icon indicates the difference in significance between pH/temperatures for the same period of interest. The statistics used was One-Way ANOVA with ⋆ *p* < 0.05. The values were expressed in mean ± SEM, representing *n* = 3 for each group. SEM is lesser than 1% of mean value for the experiment set (error bars are not evident, as they lie within the area of the symbol). The statistics to evidence the difference between immobilized and crude extract are described in the text.

Thermal and pH stabilities of *B. bassiana* and *F. oxysporum* lipases were compared before and after immobilization. The extract of *B. bassiana* lipase, (Figure 3a), presented an improvement in thermostability after immobilization (Figure 3b) at 40 °C, 50 °C, and 60 °C. Its relative activity was approximately twice as high as the initial, up to 24 and 48 h of incubation (One-Way ANOVA *p* < 0.05).

It should be noted that the relative activity, of both, extract and derivative, showed an increase of this parameter during the experiment. There was a gain in the extract activity in the first 5 h, and the derivative demonstrated activity above the 100% after 1 h of experiment at 40–60 °C (One-Way ANOVA followed by Tukey post-hoc *p* < 0.05). An explanation for this is the greater diffusion and separation of aggregates in the derivative with increasing temperature, which may be the result of a higher number of enzyme-substrate contacts in the assay. It was also observed that immobilization triggered a loss in stability at 70 °C, which may be explained by an interaction between octyl-lipase. The lipase immobilization on hydrophobic support occurs with conformational changes over the enzyme lid promoting a hyperactivation. This probably causes the catalytic site exposure and interactions within the enzyme (which are important in maintaining the native structure of the protein) to the denaturing effects in aqueous solution (like ions, hydrogen-bonding), thus, apparently, making *B. bassiana* lipase more sensitive to higher temperatures [26,52].

The immobilization also influenced its pH stability. The pH stability studies were performed at 40 °C, which was the temperature that *B. bassiana* lipase showed higher thermostability, in both, extract and octyl derivative. Therefore, there should be a concern with the interpretation of pH stability results, where it should be ascertained whether the measure of stability gain was due to a buffer effect by immobilization or thermostability gain. It could be thought that the immobilization promoted greater stability at a given pH value, since it was observed that the relative activity of the derivative was greater than the non-immobilized. However, this apparent increase might be from the thermostability after immobilization, not from buffering modifications by immobilization. It was observed that *B. bassiana* extract presented higher stability in pH 6.0 (Figure 3c), while the other pH values promoted loss in activity. The immobilization on octyl promoted a higher stability at pH 5.0 and 6.0, up to 48 h of incubation compared to the lipase extract (Figure 3c,d). Immobilization alters the interaction and the charges of backbone side chains, which may result in tridimensional structures capable of resisting hydrogen ionic strength [53,54].

### 2.4. Effect of Octyl Immobilization on F. oxysporum Lipase Stability

**Figure 4:**
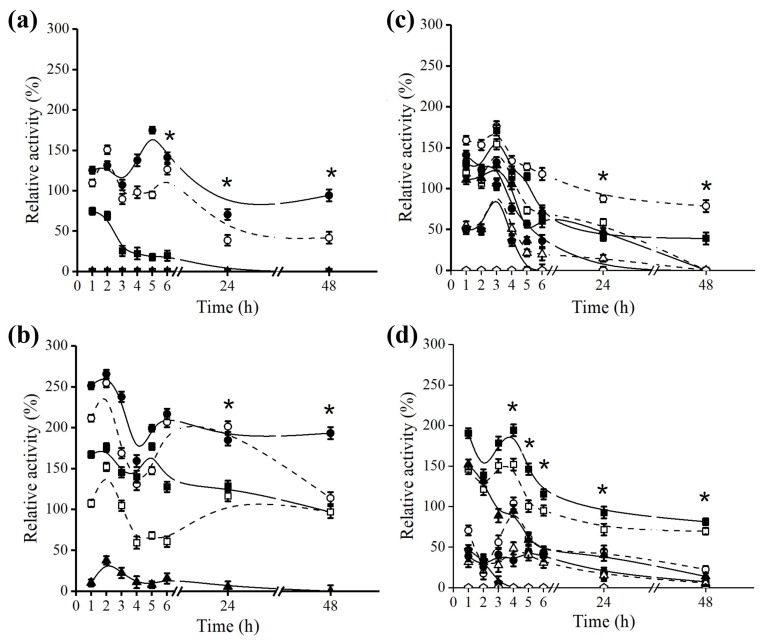
Thermal and pH stabilities of *F. oxysporum* lipase: Thermal stability of crude extract (**a**) and derivative (**b**). pH stabilities of crude extract (**c**) and derivative (**d**). Thermal inactivation was performed in McIlvaine buffer pH 7.0 at the following temperatures; (●) 30 °C; (○) 40 °C; (■) 50 °C; (□) 60 °C and (▲) 70 °C. The pH stability was performed at 40 °C with McIlvaine buffer (pH 3–8) and 200 mM glycine buffer (pH 9 and 10); (●) pH 3.0; (○) pH 4.0; (■) pH 5.0; (□) pH 6.0; (▲) pH 7.0; (△) pH 8.0; (♦) pH 9.0 and (◊) pH 10.0. Lipase activity was obtained by 0.003% *p*NPP assay and relative activity was expressed according to the initial activity of the derivative prior to incubation at different pH and temperatures at several time intervals. The 100% of relative activity was considered as 440 U of *F. oxysporum* lipase activity immobilized on Octyl measured at the beginning of experiment. Other specifications are described in Section 3.8 and Section 3.9. The symbol (⋆) above an icon indicates the difference in significance between pH/temperatures for the same period of interest. The statistics used was One-Way ANOVA with ⋆ *p* < 0.05. The values were expressed in mean ± SEM, representing *n* = 3 for each group. SEM is less than 1% of mean value for the experiment set (error bars are not evident, as they lie within the area of the symbol). The statistics to evidence the difference between immobilized and crude extract are described in the text.

The extract of *F. oxysporum* showed higher stability at 30 °C and 40 °C remaining at 90% and 50%, respectively, up to 48 h of incubation (One-Way ANOVA followed by Tukey post-hoc *p* < 0.05) (Figure 4a). Therefore, the octyl derivative showed an increase in thermal stability in the range of 30−70 °C compared to the non-immobilized lipase (One-Way ANOVA *p* < 0.05) (Figure 4b). This is a characteristic of *F. oxysporum* lipase over *B. bassiana*; the immobilization increased the thermostability of both enzymes, but only the lipase of *F. oxysporum* remained with higher relative activity in a broader temperature range with almost no loss in relative activity at 30–50 °C.

The pH stability experiments for *F. oxysporum* extract and derivative were done at 40 °C. The crude extract showed higher stability at pH 4.0, with almost no loss in relative activity, followed by pH 5.0, which held almost 50% of relative activity, up to 48 h of experiment (Figure 4c). The octyl derivative showed a gain in stability at pH 5.0 and 6.0, but a loss of approximately 50% at pH 4.0 compared to crude extract (One-Way ANOVA followed by Tukey post-hoc *p* < 0.05) (Figure 4d).

### 2.5. Desorption and Identification of Lipases

**Figure 5:**
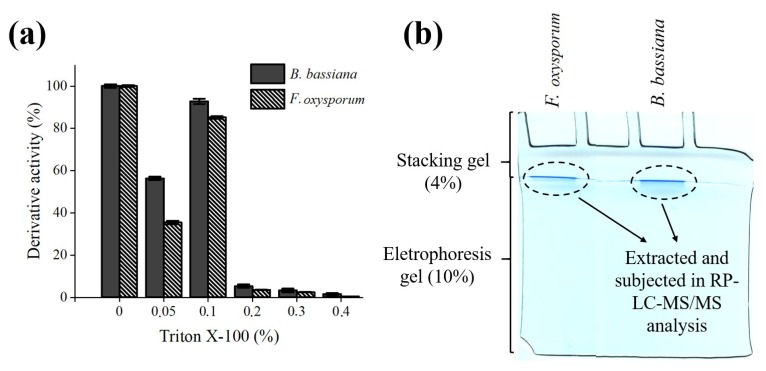
Derivative activity and lipase identification: (**a**) Profiles of *B. bassiana* and *F. oxysporum* derivatives in the presence of different concentrations of Triton X-100 (0.05; 0.1; 0.2; 0.3 and 0.4%). The enzymatic activity in the supernatant desorption was monitored by the 0.003% *p*NPP assay. The 100% of activity was considered as 440 U for Fo and 940 U for Bb. (**b**) SDS-PAGE for lipase desorption fraction of the supernatant corresponding to the concentration of 0.1% Triton X-100. The SDS-PAGE gel was stained with colloidal Coomassie. The values are expressed in mean ± SEM, with *n* = 3.

In order to identify the lipases that would be responsible for the subsequent hydrolysis of Açaí and Buriti oils, as well as to know the binding force of the enzyme with the support, increasing concentrations of Triton X-100 were used to achieve the desorption of the enzyme. A desirable aspect in industrial processes is the formation of a strong bond between the enzyme and the matrix, which allows the reuse of the catalyst and prevents the mixing of the enzyme with the products of reaction [27]. It was possible to reach almost 100% desorption of the enzyme at a concentration of 0.1% Triton X-100 for both, *F. oxysporum* and *B. bassiana*. In addition, the assays to promote desorption at a concentration of 0.05% Triton X-100, led to 59% for the lipase of *F. oxysporum* and 38% for *B. bassiana.* Other concentrations of Triton X-100 (0.2%, 0.3%, and 0.4%) evaluated did not show lipase activity in the supernatant desorbed, because at this concentration of Triton X-100 the enzyme activity is affected (Figure 5a). The enzymes studied in this work showed good resistance in the presence of detergents. For this reason, they can be used in industrial processes that employ concentrations up to 0.1% of Triton X-100. 

The supernatants corresponding to 0.1% Triton X-100 were concentrated to 20 μL and subsequently those volumes were applied to a polyacrylamide concentrator gel (20 ng·mL^−1^ of protein) (Figure 5b). After the proteins had run approximately 3 mm in the concentrator gel and before entering the separator gel, the run was stopped and the bands of the gel corresponding to the unseparated proteins were cut into small fragments. These gel fragments were digested with trypsin and later used in the identification of the proteins present in the desorbed supernatant by mass spectrometry and further, comparing them with uniprot-fungi.fasta database. In the case of *F. oxysporum,* two lipases were identified by RP-LC-MS/MS analysis and protein databases: lip1, MW (kDa): 36.5 and lip2 MW (kDa): 48.1 (Table 2). For *B. bassiana*, it was possible to identify a single lipase lip, MW (kDa): 52.9 (Table 2). For both fungi, the identified peptides, corresponding to each lipase described in Table 2, a high score was observed. These results are also in agreement with the bands obtained in the zymogram, which had molecular weights close to the lipases identified in the analyzes of RP-LC-MS/MS. The remaining spots identified in the zymogram corresponding to each of the fungi studied, which could not be detected in the spectrometric analysis, could be related to the many hydrophobic characters of the lipases; allowing some to bind with greater strength and stability to the octyl supports, enabling later identification by mass spectrometry.

Database search was performed against uniprot-fungi.fasta in Proteomic Discovery 1.4 software (Thermo Scientific, Waltham, MA, USA). Other specifications are described in Section 3.10.

### 2.6. Hydrolysis of Açaí and Buriti Oils by Lipase Immobilized on Octyl-Sepharose

Fo-octyl and Bb-octyl derivatives coated with PEI were used for the hydrolysis of the Açaí and Buriti oils. For the hydrolysis assay, 1 g of the derivatives was used in the presence of the diluted Açaí or Buriti oils in a biphasic system of cyclohexane and Tris-HCl buffer, pH 7.0. The formation of oleic and linoleic acid, products of hydrolysis were accompanied by absorbance at 215 nm in HPLC-UV. In order to correlate the absorbance data obtained in HPLC analysis with the concentration values of each of the analyzed products, standard linoleic and oleic acid curves were constructed (Figure S1). 

**Figure 6:**
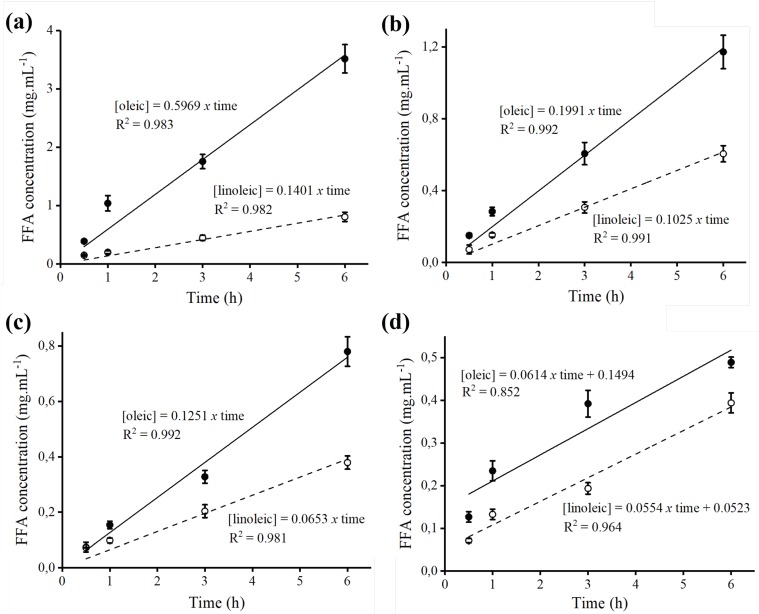
Hydrolysis of Açaí and Buriti oils: Hydrolysis using Bb-octyl derivative and Açaí (**a**) and Buriti (**b**) oils. Hydrolysis using Fo-octyl derivative and Açaí (**c**) and Buriti (**d**) oils. Concentrations of fatty acids were monitored up to 6 h of hydrolysis by RP-HPLC and were determined according to standard curves of oleic and linoleic acids. Mobile phase: (75/25, *v*/*v*) acetonitrile: 10 mM Tris-OH pH 3.0, flow rate: 1 mL.min^-1^, injected sample: 100 μL of organic phase at different times of hydrolysis previously diluted in acetonitrile. Oleic acid (●) and linoleic acid (○) produced in the hydrolysis. The values are expressed in mean ± SEM, representing *n* = 3 for each group. SEM is lesser than 1% of mean value for the experiment set (error bars are not evident, as they lie within the area of the symbol).

An interesting aspect to be debated is that an almost linear increase in the concentration of oleic and linoleic acid was observed in relation to the time of hydrolysis of Açaí and Buriti oils, by Bb-octyl and Fo-octyl derivatives (Figure 6). However, when the hydrolysis of both oils was extended for a period of 24 h, the formation of the products was reduced by 50% for both derivatives (results not shown). This fact may be related to the decrease in lipase stability, since the mechanical agitation of the sustained hydrolysis reaction medium over a prolonged period may promote unbinding of the enzyme to the octyl support, leading to a loss in enzyme stability and hydrolysis efficiency [55].

Likewise, the productivity indexes (amount in mg of fatty acid produced per hour of hydrolysis) of oleic and linoleic acids were calculated demonstrating a higher productivity index for oleic acid in relation to linoleic acid, for both derivatives and hydrolyzed oils (Table 3). A possible explanation for this fact is that oleic acid is present in a greater proportion in the composition of these oils in comparison to linoleic acid. This factor may favor a greater release of oleic acid in the organic phase. Additionally, our results are in accordance with data shown by other authors, showing the preference of lipase for the cleavage of triacylglycerides at the oleic acid chain [56,57]. Comparing both derivatives, Bb immobilized lipase demonstrated higher capacity to produce fatty acids from Açaí and Buriti oils, in relation to the Fo immobilized lipase. 

Mobile phase: (75/25, *v*/*v*) acetonitrile: 10 mM Tris-OH pH 3.0, flow rate: 1 mL·min^−1^, injected sample: 100 μL of organic phase previously diluted in acetonitrile. The productivity index (mg·h^-1^) was determined using Equation (3). The values are expressed by mean ± SEM, with *n* = 3.

## 3. Materials and Methods

### 3.1. Microorganisms and Oils

*F. oxysporum* and *B. bassiana* used in this work were isolated from soil samples in the region of Ribeirão Preto (São Paulo-Brazil). Samples are deposited at the Fungal Collection at The Microbiology and Cell Biology Laboratory of the Faculty of Philosophy, Sciences and Letters of Ribeirão Preto, University of São Paulo, Brazil. *F. oxysporum* was maintained on slants with potato dextrose agar [58] medium and *B. bassiana* in solid Khanna medium [59] with 1% glucose. Both fungi were grown at 30 °C for seven days.

Twenty oils and butters commercially obtained were tested as carbon source, most of them from the Brazilian Amazon Forest: Buriti (*M. flexuosa*); Açaí (*E. oleracea* Martius); Pracaxi (*Pentaclethra macroloba*); Babaçu (*Attalea speciosa*); Cupuaçu (*Theobroma grandiflorum*); Ojon (*Elaeis Oleifera*); Muru muru (*Astrocaryum murumuru*); Tucumã (*Astrocaryum aculeatum*); Brazil nut (*Bertholletia excels*); Andiroba (*Carapa guianensis*); Grape seed (*Vitis vinifera* L.); Passion fruit (*Passiflora edulis*); Chia (*Salvia hispanica* L.); Ucuuba (*Virola surinamensis*); Canola (*Brassica napus*); Bacuri (*Platonia insignis*); Peanut (*Arachis hypogaea* L.); Avocado (*Persea americana*); Fish (*Sardinella brasiliensis*); and Coffee (*Coffea* sp.).

### 3.2. Carbon Source Selection and Lipase Production

A volume of 1 mL (10^6^ spores from *F. oxysporum* and *B. bassiana*) previously ressuspended in distilled water, was separately inoculated into Erlenmeyer flasks of 125 mL containing 25 mL of Adams [60] liquid medium and 0.5 mL of the different oils and butters listed in Section 2.1. Cultures were placed in a shaker at 100 rpm, 30 °C for 72 h. After the required period, the mycelia of the fungi were separated from the liquid media by vacuum filtration and Whatman^®^ filter paper number 1, the crude filtrate obtained was considered as a source of extracellular lipase activity.

### 3.3. Enzyme Activity Assay

The enzymatic activity was kinetically monitored by the increase in absorbance readings at 405 nm, produced by the formation of 4-nitrophenolate in alkaline medium due to the hydrolysis of *p*-nitrophenyl palmitate (*p*NPP) (Sigma Aldrich) by lipase. The assay was conducted by the addition of 0.01 mL of enzyme solution or suspension (derivative plus enzyme solution) to a 0.09 mL mixture containing McIlvaine buffer pH 6.0, 12.0 mM *p*NPP (previously dissolved in isopropyl alcohol), 0.1% (*v*/*v*) Triton X-100 (Sigma-Aldrich), 0.05% (*m*/*v*) gum arabic (Sigma-Aldrich) for 5 min at 55 °C. The assay was stopped through the addition of a saturated sodium tetraborate solution at 1:1 ratio. Finally, 200 μL of the mixture were transferred to a 96-well Enzyme-Linked Immunosorbent Assay (ELISA) plate and the absorbance was measured on a SpectraMax (Molecular Devices) reader. By convention, a (U) unit of enzyme activity was defined as the amount of enzyme required to hydrolyze 1 μmol of *p*NPP per minute under the conditions described above.

### 3.4. Protein Measurement

Proteins were measured by the Lowry method [61], using bovine serum albumin (BSA) as standard. Protein values were expressed as mg of protein per mL. Specific activity was expressed as U·mg^−1^ of protein.

### 3.5. Protein Electrophoresis in Polyacrylamide Gel under Semi-Denaturing Conditions

Electrophoresis in semi-denaturing conditions (Sodium Dodecyl Sulfate Polyacrylamide Gel Electrophoresis- SDS-PAGE) was performed similarly to the methodology described by Laemmli (1970) [62]. The semi-denaturing character is conferred because the run buffer did not contain β-mercaptoethanol and the amount of SDS present was lower than the original methodology (0.01% SDS was used in this methodology), in addition, the samples were not boiled and after the run, buffer was added [63]. The gels were stained with a silver solution [64].

### 3.6. Zymogram

In order to visualize the lipolytic activity, the zymogram was developed as described by Kwon et al. (2011) [63]. The assay led to the formation of complexes that were visualized as dark spots on the polyacrylamide gel.

### 3.7. Immobilization of Lipase on Octyl-Sepharose Support and Coating with Polyethyleneimine (PEI)

Two grams of octyl-sepharose were added to 2.5 mL of *B. bassiana* crude extract and 40 mL of *F. oxysporum* extract obtained as described in Section 2.2 (20 U of total activity per gram of support) in sodium phosphate buffer 50 mM, pH 7.0. This mixture was kept under a roller, stirring at 4 °C. Periodically, aliquots of the suspension and supernatant were taken and then subjected to lipase activity assays. The activity value of the derivative from *B. bassiana* (Bb adsorbed) and *F. oxysporum* (Fo adsorbed) corresponded to the subtraction between the value obtained from the suspension and the supernatant. The enzymatic activities at each instant were compared to the initial immobilization time, and the values obtained from activity as residual activity were described. After 24h, the derivatives were filtered, exhaustively washed with distilled water, and stored at 4 °C, until use. Immobilization yield (Y) and relative activity of octyl derivative were calculated according to Equations (1) and (2), respectively:

(1) $$Y\;(\%)\;=\;\frac{A-B}{A}\;\times \;100$$

(2) $$\mathrm{Relative}\;\mathrm{activity}\;\mathrm{of}\;\mathrm{octyl}\;\mathrm{derivate}\;(\%)\;=\;\frac{C}{A\times Y}\;\times \;100$$

In which *A* is the total activity of the solution offered to immobilization (40 U), *B* is the activity of the supernatant after 24 h of immobilization reaction, and C is the activity present in octyl derivative after immobilization, expressed in U per gram of octyl used in the reaction. 

Subsequently, 1 g of the obtained derivatives (Fo-octyl and Bb-octyl) was coated with 10 mg of polyethyleneimine (PEI) 25 kDa, pre-diluted in 10 mL of sodium phosphate buffer, 25 mM, pH 7.0. Derivatives coated with PEI were placed on a roller shaking at 4 °C, overnight. After this period, the derivatives were filtered and washed several times with distilled water.

### 3.8. Thermal Inactivation and pH Stability of the Crude Extract and Derivative

Thermal inactivation of the derivatives (Fo-octyl and Bb-octyl), as well as the crude extracts of Bb and Fo were evaluated. For Fo-octyl and crude extract of Fo the range of temperature for carrying out was 30–70 °C and for Bb-octyl and crude extract of Bb the range of temperature used was 40–80 °C. For the Fo or Bb, 0.5 mL of the crude extracts was diluted in 0.5 mL McIlvaine buffer pH 7.0 and for the derivate, 40 mg was diluted in the same buffer. Both crude extracts and derivatives were kept substrate-free and in a thermostatic bath at the pre-determined temperatures and times for the assay processing. Periodically, 10 μL aliquots were subjected to *p*NPP hydrolysis.

The pH stability of the derivatives (Fo-octyl and Bb-octyl) and the crude extracts were evaluated at 40 °C after several incubation times in buffers with pH ranging from 3–10. The buffers used were: McIlvaine buffer (pH of 3–8) and 200 mM glycine buffer pH 9–10.

For the crude extracts, 0.5 mL of the Fo or Bb was diluted in 0.5 mL of each buffer used in the experiments. In the case of the derivatives, 40 mg was diluted with the same buffers. The derivatives and crude extracts were kept in suspension and substrate-free. Periodically, 10 μL aliquots were subjected to *p*NPP hydrolysis. 

In both experiments, the results were expressed in Residual Activity (%) and 100% was considered the value of the enzymatic activity before the incubation periods started. 

### 3.9. Desorption of Lipase from Octyl-Sepharose Support

Octyl-sepharose derivatives obtained (Section 2.7) were suspended in 25 mL of 5 mM sodium phosphate buffer, pH 7.0. Subsequently, increasing concentrations (0–0.4%) of Triton X-100 were added to the derivatives and incubated for 45 min at room temperature. After that, aliquots of the derivatives and supernatant were withdrawn at several times to monitor the enzymatic activity. An assay blank with soluble enzyme was subjected to the same treatment to detect any possible effect of Triton X-100 on the enzymatic activity.

### 3.10. Reverse Phase-Liquid Chromatography RP-LC-MS/MS Analysis

The supernatant fraction of each of the derivatives corresponding to the concentration of 0.1% Triton X-100 (Section 2.9) was concentrated in a Speedvac to a volume of 20 μL. Afterwards, the 20 µL was suspended in a volume, up to 50 µL of sample buffer, and then, applied to 1.2-cm wide wells of a conventional SDS-PAGE gel (0.75 mm-thick, 4% stacking, and 10% resolving). Then the run was stopped as soon as the front entered 3 mm into the resolving gel, so that the whole proteome became concentrated in the stacking/resolving gel interface. The unseparated protein bands were visualized by colloidal Coomassie staining, excised, cut into cubes (2 × 2 × 0.75 mm), and placed in 0.5 mL microcentrifuge tubes [64]. The gel pieces were digested with trypsin (Promega, Madison, WI), and analyzed by RP-LC-MS/MS (Thermo Scientific, Waltham, MA, USA) in an Easy-nLC II system coupled to an ion trap LTQ-Orbitrap-Velos-Pro hybrid mass spectrometer (Thermo Scientific). 

Peptide identification from raw data was carried out using the SEQUEST algorithm (Proteome Discoverer 1.4, Thermo Scientific). Database search was performed against uniprot-fungi.fasta. The following constraints were used for the searches: tryptic cleavage after Arg and Lys, up to two missed cleavage sites, and tolerances of 10 ppm for precursor ions and 0.8 Da for MS/MS fragment ions and the searches were performed allowing optional Met oxidation and Cys carbamidomethylation. Search against decoy database (integrated decoy approach) using false discovery rate (FDR) < 0.01.

### 3.11. Hydrolysis of Açaí and Buriti Oils

The hydrolysis of Buriti and Açaí oils was carried out in an organic/aqueous two-phase system. Açaí or Buriti oils (0.5 mL) were added to a solution of 5 mL of cyclohexane and 5 mL of 0.1 M Tris-HCl, pH 7.0. Subsequently this mixture was incubated in a reactor for 30 min, at 25 °C, and 150 rpm to facilitate the solubility of the oils in the water. After 30 min of incubation, the reaction was started by the addition of 1 g of the derivatives Fo-octyl or Bb-octyl. Concentrations of free fatty acids were evaluated over several periods of time using HPLC-UV. In this experiment “t_0_” was considered the solution prior to the derivative addition.

### 3.12. Analysis of Free Fatty Acids (Omega-6 and 9) by HPLC-UV

After several periods of time, aliquots of 0.1 mL of the organic phase were withdrawn and dissolved in 0.8 mL of acetonitrile, of which 0.1 mL was used for analysis of free fatty acids on HPLC. The organic phase was easily separated from the aqueous phase when stirring of the biphasic system was discontinued. The produced unsaturated fatty acids were analyzed by RP-HPLC [Spectra Physic SP 100 coupled with a Spectra Physic SP 8450 UV detector (Spectra Physics, Santa Clara, CA USA)] using a Kromasil C8 column (15 cm × 0.4 cm). The products were eluted at an isocratic flow of 1.0 mL·min^−1^ using 10 mM Tris-OH buffer pH 3.0 (75:25, *v*/*v*) acetonitrile and the UV detection performed at 215 nm. The fatty acids produced by the hydrolysis of the Açaí and Buriti oils by each lipase were compared with the corresponding pure commercial standards. The production index (amount of fatty acid formed per hour of hydrolysis of Açaí and Buriti oils) of oleic (omega-9) and linoleic (omega-6) fatty acids was calculated according to the following formula (Equation (3)).

(3) PI=Vol × a

In which “PI” is the productivity index, expressed in mg·h^−1^, “Vol” is the total volume of the hydrolysate reaction medium (11.2 mL) and “a” is the slope of the oleic (omega-9) or linoleic acid (omega-6) concentration curve.

### 3.13. Statistical Analysis 

The data are expressed as mean ±SEM. The differences observed between the several experimental values were analyzed by one-way analysis of variance (ANOVA) followed by the parametric Tukey test to compare multiple values. All analyses were performed using Statistica 8.0 software Copyright© StatSoft (Tulsa, OK, USA). Statistical significance was set at *p* < 0.05.

## 4. Conclusions

In this work the authors, successfully developed a bioprocess for the production of omegas-6 and 9 from the hydrolysis of Buriti and Açaí oils by immobilized lipases. The filamentous fungi *B. bassiana* and *F. oxysporum* were grown on several Amazon native oils and butters. The selected Açaí oil and Bacuri butter were chosen as carbon source and substrate to produce omegas. Both enzymatic extracts were immobilized on octyl-sepharose. The immobilization resulted in the hyperactivation and stabilization of lipases produced by both microorganisms. There were two lipases identified for *F. oxysporum* and one for *B. bassiana* in the derivative. The HPLC analysis showed the production of omegas-6 and 9 after the hydrolysis of Açaí and Buriti oils by both immobilized extracts of lipase.

*B. bassiana* is widely known as an entomopathogenic fungus, and it is already commonly used in agriculture, to the benefit of crops. This paper demonstrated that its lipase could produce omegas-6 and 9, inputs of medical and nutritional importance, which enhances the importance of this fungus. As for the oils used as hydrolysis substrates, Açaí showed great potential in obtaining these omegas. Açaí and its derivatives are part of the Brazilian diet and move the economy of small farmers, benefiting local social development and the economic funds of the country.

Among the future perspectives of this work, the refinement of the methods aiming to obtain omegas from Brazilian oils is expected using *B. bassiana* and *F. oxysporum* lipases that demonstrated high catalytic potential, even in wild strains. This characteristic makes the lipases produced by these fungi different from those produced by other microorganisms. Therefore, optimization studies on hydrolysis and obtainment of mutant strains, with high potential for the production of these lipases in particular, are necessary to maximize this process, ensuring economic and sustainable enrichment of the fatty acid market.

## Figures and Tables

**Table 1:** Parameters of *B. bassiana* and *F. oxysporum* lipase immobilization on octyl-sepharose.

Extract	Yield from Supernatant (%)	Relative Activity of Octyl Derivative (%)	Octyl Derivative Specific Activity (U·g^−1^)
*B. bassiana*	81.78 ± 0.75	2350 ± 22.17	470 ± 13.46
*F.oxysporum*	78.27 ± 0.67	1100 ± 11.32	220 ± 18.32

**Table 2:** Identification of *B. bassiana* and *F. oxysporum* lipase by RP-LC-MS/MS analysis.

Lipase	Accession	Score	Coverage (%)	No. Peptides	MW (kDa)	Calc. *pI*
*F. oxysporum lip 1*	A0A0C4DHT8	873.70	33.82	8	36.5	6.86
*F. oxysporum lip 2*	A0A0D2YK59	5.08	5.13	2	48.1	5.88
*B. bassiana lip*	J4VUX9	825.96	21.23	10	52.9	6.57

**Table 3:** Productivity index of oleic and linoleic acids by Bb-octyl and Fo-octyl derivatives.

Parameter	Derivative	Açaí		Buriti	
		Oleic Acid	Linoleic Acid	Oleic Acid	Linoleic Acid
Productivity index (mg·h^−1^)	Bb-octyl	6.68 ± 0.23	1.57 ± 0.09	2.22 ± 0.18	1.15 ± 0.02
	Fo-octyl	1.41 ± 0.17	0.73 ± 0.12	1.06 ± 0.02	0.75 ± 0.01

## Supplementary Materials

The following are available online, Figure S1: UV response of linoleic and oleic acids at different concentrations.

## References

[1] Zielińska A., Nowak I. (2014). Fatty Acids in Vegetable Oils and Their Importance in Cosmetic Industry. Chemik.

[2] Bhardwaj K., Verma N., Trivedi R.K., Bhardwaj S., Shukla N. (2016). Significance of Ratio of Omega-3 and Omega-6 in Human Health with Special Reference to Flaxseed Oil. Int. J. Biol. Chem..

[3] Omega-3, 6, and 9 and How They Add Up. https://www.uccs.edu/healthcircle/sites/healthcircle/files/inline-files/Omega-3_6_and_9_Fats.pdf.

[4] Asif M. (2011). Health Effects of Omega-3,6,9 Fatty Acids: *Perilla frutescens* is a Good Example of Plant Oils. Orient. Pharm. Exp. Med..

[5] Patterson E., Wall R., Fitzgerald G.F., Ross R.P., Stanton C. (2012). Health Implications of High Dietary Omega-6 Polyunsaturated Fatty Acids. J. Nutr. Metab..

[6] Kapoor R., Huang Y.-S. (2006). Gamma Linolenic Acid: An Antiinflammatory Omega-6 Fatty Acid. Curr. Pharm. Biotechnol..

[7] Derbyshire E. (2017). Do Omega-3/6 Fatty Acids Have a Therapeutic Role in Children and Young People with ADHD?. J. Lipids.

[8] Gultekin G., Sahin H., Inanc N., Uyanik F., Ok E. (2014). Impact of Omega-3 and Omega-9 Fatty Acids Enriched Total Parenteral Nutrition on Blood Chemistry and Inflammatory Markers in Septic Patients. Pakistan J. Med. Sci..

[9] Johnson M., Bradford C. (2014). Omega-3, Omega-6 and Omega-9 Fatty Acids: Implications for Cardiovascular and Other Diseases. J. Glycomics Lipidomics.

[10] Kien C.L., Bunn J.Y., Tompkins C.L., Dumas J.A., Crain K.I., Ebenstein D.B., Koves T.R., Muoio D.M. (2013). Substituting Dietary Monounsaturated Fat for Saturated Fat is Associated with Increased Daily Physical Activity and Resting Energy Expenditure and with Changes in Mood. Am. J. Clin. Nutr..

[11] Kim E., Ko H.J., Jeon S.J., Lee S., Lee H.E., Kim H.N., Woo E.R., Ryu J.H. (2016). The Memory-Enhancing Effect of Erucic Acid on Scopolamine-Induced Cognitive Impairment in Mice. Pharmacol. Biochem. Behav..

[12] De Rosso V.V., Mercadante A.Z. (2007). Identification and Quantification of Carotenoids, by HPLC-PDA-MS/MS, from Amazonian Fruits. J. Agric. Food Chem..

[13] Rufino M.S.M., Alves R.E., de Brito E.S., Pérez-Jiménez J., Saura-Calixto F., Mancini-Filho J. (2010). Bioactive Compounds and Antioxidant Capacities of 18 Non-Traditional Tropical Fruits from Brazil. Food Chem..

[14] Pereira Lima R., Souza da Luz P.T., Braga M., dos Santos Batista P.R., Ferreira da Costa C.E., Zamian J.R., Santos do Nascimento L.A., da Rocha Filho G.N. (2017). Murumuru (*Astrocaryum murumuru* Mart.) Butter and Oils of Buriti (*Mauritia flexuosa* Mart.) and Pracaxi (*Pentaclethra macroloba* (Willd.) Kuntze) Can be Used for Biodiesel Production: Physico-chemical Properties and Thermal and Kinetic Studies. Ind. Crops Prod..

[15] Albuquerque M.L.S., Guedes I., Alcantara P., Moreira S.G.C., Barbosa Neto N.M., Correa D.S., Zilio S.C. (2005). Characterization of Buriti (*Mauritia flexuosa* L.) Oil by Absorption and Emission Spectroscopies. J. Braz. Chem. Soc..

[16] Nascimento R.J.S., Couri S., Antoniassi R., Freitas S.P. (2008). Composição em Ácidos Graxos do Óleo da Polpa de Açaí Extraído com Enzimas e com Hexano. Rev. Bras. Frutic..

[17] Darnet S.H., Silva L.H.M., Rodrigues A.M.C., Lins R.T. (2011). Nutritional Composition, Fatty Acid and Tocopherol Contents of Buriti (*Mauritia flexuosa*) and Patawa (*Oenocarpus bataua*) Fruit Pulp from the Amazon Region. Ciência e Tecnol. Aliment..

[18] Fernández-Lorente G., Pizarro C., López-Vela D., Betancor L., Carrascosa A.V., Pessela B., Guisan J.M. (2011). Hydrolysis of Fish Oil by Lipases Immobilized Inside Porous Supports. J. Am. Oil Chem. Soc..

[19] Shimada Y., Sugihara A., Tominaga Y. (2001). Enzymatic Purification of Polyunsaturated Fatty Acids. J. Biosci. Bioeng..

[20] Singh A.K., Mukhopadhyay M. (2012). Overview of Fungal Lipase: A Review. Appl. Biochem. Biotechnol..

[21] Peirce S., Tacias-Pascacio V., Russo M., Marzocchella A., Virgen-Ortíz J., Fernandez-Lafuente R. (2016). Stabilization of *Candida antarctica* Lipase B (CALB) Immobilized on Octyl Agarose by Treatment with Polyethyleneimine (PEI). Molecules.

[22] Hasan F., Shah A.A., Hameed A. (2006). Industrial Applications of Microbial Lipases. Enzyme Microb. Technol..

[23] Zdarta J., Meyer A., Jesionowski T., Pinelo M. (2018). A General Overview of Support Materials for Enzyme Immobilization: Characteristics, Properties, Practical Utility. Catalysts.

[24] Andualema B., Gessesse A. (2012). Microbial Lipases and Their Industrial Applications: Review. Biotechnology.

[25] Palomo J.M., Muñoz G., Fernández-Lorente G., Mateo C., Fernández-Lafuente R., Guisán J.M. (2002). Interfacial Adsorption of Lipases on Very Hydrophobic Support (Octadecyl–Sepabeads): Immobilization, Hyperactivation and Stabilization of the Open Form of Lipases. J. Mol. Catal. B Enzym..

[26] Manoel E.A., dos Santos J.C.S., Freire D.M.G., Rueda N., Fernandez-Lafuente R. (2015). Immobilization of Lipases on Hydrophobic Supports Involves the Open Form Of the Enzyme. Enzyme Microb. Technol..

[27] Pereira M.G., Facchini F.D.A., Filó L.E.C., Polizeli A.M., Vici A.C., Jorge J.A., Lorente G.F., Pessela B.C., Guisan J.M., Polizeli M.L.T.M. (2015). Immobilized Lipase From *Hypocrea pseudokoningii* on Hydrophobic and Ionic Supports: Determination of Thermal and Organic Solvent Stabilities for Applications in the Oleochemical Industry. Process Biochem..

[28] Palomo J.M., Fernandez-Lorente G., Mateo C., Ortiz C., Fernandez-Lafuente R., Guisan J.M. (2002). Modulation Of The Enantioselectivity of Lipases Via Controlled Immobilization and Medium Engineering: Hydrolytic Resolution of Mandelic Acid Esters. Enzyme Microb. Technol..

[29] Fernández-Lorente G., Betancor L., Carrascosa A.V., Palomo J.M., Guisan J.M. (2012). Modulation of the Selectivity of Immobilized Lipases by Chemical and Physical Modifications: Release of Omega-3 Fatty Acids from Fish Oil. J. Am. Oil Chem. Soc..

[30] Mateo C., Palomo J.M., Fernandez-Lorente G., Guisan J.M., Fernandez-Lafuente R. (2007). Improvement of Enzyme Activity, Stability and Selectivity Via Immobilization Techniques. Enzyme Microb. Technol..

[31] Rueda N., Dos Santos J.C.S., Torres R., Ortiz C., Barbosa O., Fernandez-Lafuente R. (2015). Improved Performance of Lipases Immobilized on Heterofunctional Octyl-Glyoxyl Agarose Beads. RSC Adv..

[32] Virgen-Ortíz J.J., Tacias-Pascacio V.G., Hirata D.B., Torrestiana-Sanchez B., Rosales-Quintero A., Fernandez-Lafuente R. (2017). Relevance of Substrates and Products on the Desorption of Lipases Physically Adsorbed on Hydrophobic Supports. Enzyme Microb. Technol..

[33] Guajardo N., Bernal C., Wilson L., Cabrera Z. (2015). Selectivity of R-α-monobenzoate Glycerol Synthesis Catalyzed by *Candida antarctica* Lipase B Immobilized on Heterofunctional Supports. Process Biochem..

[34] Albuquerque T.L.D., Rueda N., dos Santos J.C.S., Barbosa O., Ortiz C., Binay B., Özdemir E., Gonçalves L.R.B., Fernandez-Lafuente R. (2016). Easy Stabilization of Interfacially Activated Lipases Using Heterofunctional Divinyl Sulfone Activated-Octyl Agarose Beads. Modulation of The Immobilized Enzymes by Altering Their Nanoenvironment. Process Biochem..

[35] Rueda N., dos Santos C.S., Rodriguez M.D., Albuquerque T.L., Barbosa O., Torres R., Ortiz C., Fernandez-Lafuente R. (2016). Reversible Immobilization of Lipases on Octyl-Glutamic Agarose Beads: A Mixed Adsorption that Reinforces Enzyme Immobilization. J. Mol. Catal. B Enzym..

[36] Rueda N., Albuquerque T., Bartolome-Cabrero R., Fernandez-Lopez L., Torres R., Ortiz C., dos Santos J., Barbosa O., Fernandez-Lafuente R. (2016). Reversible Immobilization of Lipases on Heterofunctional Octyl-Amino Agarose Beads Prevents Enzyme Desorption. Molecules.

[37] Virgen-Ortíz J.J., dos Santos J.C.S., Berenguer-Murcia Á., Barbosa O., Rodrigues R.C., Fernandez-Lafuente R. (2017). Polyethylenimine: A Very Useful Ionic Polymer in the Design of Immobilized Enzyme Biocatalysts. J. Mater. Chem. B.

[38] Velasco-Lozano S., López-Gallego F., Vázquez-Duhalt R., Mateos-Díaz J.C., Guisán J.M., Favela-Torres E. (2014). Carrier-Free Immobilization of Lipase from *Candida rugosa* with Polyethyleneimines by Carboxyl-Activated Cross-Linking. Biomacromolecules.

[39] Fernandez-Lopez L., Virgen-OrtÍz J.J., Pedrero S.G., Lopez-Carrobles N., Gorines B.C., Otero C., Fernandez-Lafuente R. (2018). Optimization of the Coating Of Octyl-CALB with Ionic Polymers to Improve Stability and Decrease Enzyme Leakage. Biocatal. Biotransformation.

[40] Zaak H., Fernandez-Lopez L., Otero C., Sassi M., Fernandez-Lafuente R. (2017). Improved Stability of Immobilized Lipases Via Modification with Polyethylenimine and Glutaraldehyde. Enzyme Microb. Technol..

[41] Bolivar J.M., Rocha-Martin J., Mateo C., Cava F., Berenguer J., Fernandez-Lafuente R., Guisan J.M. (2009). Coating of Soluble and Immobilized Enzymes with Ionic Polymers: Full Stabilization of the Quaternary Structure of Multimeric Enzymes. Biomacromolecules.

[42] Andersson M.M., Breccia J.D., Hatti-Kaul R. (2000). Stabilizing Effect of Chemical Additives Against Oxidation of Lactate Dehydrogenase. Biotechnol. Appl. Biochem..

[43] Breccia J.D., Andersson M.M., Hatti-Kaul R. (2002). The Role of Poly(Ethyleneimine) in Stabilization Against Metal-Catalyzed Oxidation of Proteins: A Case Study with Lactate Dehydrogenase. Biochim. Biophys. Acta Gen. Subj..

[44] Cabrera Z., Gutarra M.L.E., Guisan J.M., Palomo J.M. (2010). Highly Enantioselective Biocatalysts by Coating Immobilized Lipases with Polyethyleneimine. Catal. Commun..

[45] Ranjan Moharana T., Byreddy A.R., Puri M., Barrow C., Rao N.M. (2016). Selective Enrichment of Omega-3 Fatty Acids in Oils by Phospholipase A1. PLoS One.

[46] Fernández-Lorente G., Betancor L., Carrascosa A.V., Guisán J.M. (2011). Release of Omega-3 Fatty Acids by the Hydrolysis of Fish Oil Catalyzed by Lipases Immobilized on Hydrophobic Supports. J. Am. Oil Chem. Soc..

[47] Moreno M.L., Escobar J., Izquierdo-Álvarez A., Gil A., Pérez S., Pereda J., Zapico I., Vento M., Sabater L., Marina A., Martínez-Ruiz A., Sastre J. (2014). Disulfide stress: A Novel Type of Oxidative Stress in Acute Pancreatitis. Free Radic. Biol. Med..

[48] Santos R.C., Filho A.A.M., Chagas E.A., Takahashi J.A., Montero I.F., Holanda L.C., Ribeiro P.R.E., Santos G.F., Melo A.C.G.R. (2017). Chemical Characterization of Oils and Fats from Amazonian Fruits by ^1^H-NMR.

[49] Hiane P.A., Bogo D., Ramos M.I.L., Ramos Filho M.M. (2003). Carotenóides Pró-Vitamínicos A e Composição em Ácidos Graxos do Fruto e da Farinha do Bacuri (*Scheelea phalerata* Mart.). Ciência e Tecnol. Aliment..

[50] Lakshmi B.S., Kangueane P., Abraham B., Pennathur G. (1999). Effect of Vegetable Oils in the Secretion of Lipase from *Candida rugosa* (DSM 2031). Lett. Appl. Microbiol..

[51] Fernández-Lorente G., Palomo J.M., Fuentes M., Mateo C., Guisán J.M., Fernández-Lafuente R. (2003). Self-assembly of *Pseudomonas fluorescens* Lipase into Bimolecular Aggregates Dramatically Affects Functional Properties. Biotechnol. Bioeng..

[52] González-Navarro H., Bañó M.C., Abad C. (2001). The Closed/open Model for Lipase Activation. Addressing Intermediate Active Forms of Fungal Enzymes by Trapping of Conformers in Water-Restricted Environments. Biochemistry.

[53] Maiangwa J., Mohamad Ali M.S., Salleh A.B., Rahman R.N.Z.R.A., Normi Y.M., Mohd Shariff F., Leow T.C. (2017). Lid Opening and Conformational Stability of T1 Lipase is Mediated by Increasing Chain Length Polar Solvents. Peer J..

[54] Mohamad N.R., Marzuki N.H.C., Buang N.A., Huyop F., Wahab R.A. (2015). An Overview of Technologies for Immobilization of Enzymes and Surface Analysis Techniques for Immobilized Enzymes. Biotechnol. Biotechnol. Equip..

[55] Yang Q., Wang B., Zhang Z., Lou D., Tan J., Zhu L. (2017). The Effects of Macromolecular Crowding and Surface Charge on the Properties of an Immobilized Enzyme: Activity, Thermal Stability, Catalytic Efficiency and Reusability. RSC Adv..

[56] Padilha M.E.S., Augusto-Ruiz W. (2007). Hidrólise Enzimática do Óleo de Pescado. Ciência e Tecnol. Aliment..

[57] Ferreira-Dias S., Sandoval G., Plou F., Valero F. (2013). The Potential Use of Lipases in the Production of Fatty Acid Derivatives for the Food and Nutraceutical Industries. Electron. J. Biotechnol..

[58] Pereira M.G., Vici A.C., Facchini F.D.A., Tristão A.P., Cursino-Santos J.R., Sanches P.R., Jorge J.A., Polizeli M.L.T.M. (2014). Screening of Filamentous Fungi for Lipase Production: *Hypocrea pseudokoningii* A New Producer With a High Biotechnological Potential. Biocatal. Biotransform..

[59] Khanna P., Sundari S.S., Kumar N.J. (1995). Production, Isolation and Partial Purification of Xylanases from an *Aspergillus* sp.. World J. Microbiol. Biotechnol..

[60] Adams P.R. (1990). Mycelial Amylase Activities of Thermophilic Species of *Rhizomucor*, *Humicola* and *Papulaspora*. Mycopathologia.

[61] Lowry O.H., Rosebrough N.J., Farr A.L., Randall R.J. (1951). Protein Measurement with the Folin Phenol Reagent. J. Biol. Chem..

[62] Laemmli U.K. (1970). Cleavage of Structural Proteins During the Assembly of the Head of Bacteriophage T4. Nature.

[63] Kwon M.A., Kim H.S., Hahm D.H., Song J.K. (2011). Synthesis Activity-based Zymography for Detection of Lipases and Esterases. Biotechnol. Lett..

[64] Oakley B.R., Kirsch D.R., Morris N.R. (1980). A Simplified Ultrasensitive Silver Stain for Detecting Proteins in Polyacrylamide Gels. Anal. Biochem..

