# Supplementary material for: Production of Omegas-6 and 9 from the Hydrolysis of Açaí and Buriti Oils by Lipase Immobilized on a Hydrophobic Support
Source: Molecules. 2018 Nov 18;23(11):3015. doi: 10.3390/molecules23113015 (PMC6278552; doi:10.3390/molecules23113015)
Supplement: Supplementary file 1 [file molecules-23-03015-s001.pdf]

molecules

MDPI

Article

Production of Omegas-6 and 9 from the Hydrolysis of Açaí and Buriti Oils by Lipase Immobilized on a Hydrophobic Support

Malena Martínez Pérez [1], Enrico Cerioni Spiropulos Gonçalves [1], Jose Carlos Santos Salgado [2], Mariana de Souza Rocha [1], Paula Zaghetto de Almeida [1], Ana Claudia Vici [3], Juliana da Conceição Infante [1], Jose Manuel Guisán [4], Javier Rocha-Martin [4], Benevides Costa Pessela [5,6] and Maria de Lourdes Teixeira de Moraes Polizeli [1,3,*]

1 Departamento de Bioquímica e Imunologia, Faculdade de Medicina de Ribeirão Preto, Universidade de São Paulo, Ribeirão Preto, São Paulo 14049-900, Brazil; malenac@usp.br (M.M.P.); spiropulos.enrico@gmail.com (E.C.S.G.); mariana_rocha@usp.br (M.S.R.); paulazag@usp.br (P.Z.A.); julianainfante@usp.br (J.C.I.)
2 Departamento de Química, Faculdade de Filosofia, Ciências e Letras de Ribeirão Preto, Universidade de São Paulo, Ribeirão Preto, São Paulo 14049-901, Brazil; salgado@usp.br
3 Departamento de Biologia, Faculdade de Filosofia, Ciências e Letras de Ribeirão Preto, Universidade de São Paulo, Ribeirão Preto, São Paulo 14040-901, Brazil; acvici@usp.br (A.C.V.)
4 Departamento de Biocatálisis, Instituto de Catálisis y Petroleoquímica, CSIC, Campus UAM, Cantoblanco, 28049 Madrid, Spain; jmguisan@icp.csic.es (J.M.G.); javirocha@icp.csic.es (J.R.M.)
5 Departamento de Biotecnología y Microbiologia de los Alimentos, Instituto de Ciencias de la Alimentación, CIAL-CSIC, Campus UAM, Cantoblanco, 28049, Spain: b.pessela@csic.es
6 Departamento de Engenharia e Tecnologias, DET- Instituto Superior Politecnico de Tecnologias e Ciências-ISPTEC, Av. Luanda Sul, Rua Lateral Via S10, Talatona-Republica de Angola
* Correspondence: polizeli@ffclrp.usp.br; Tel.: +55-16-3315-4680

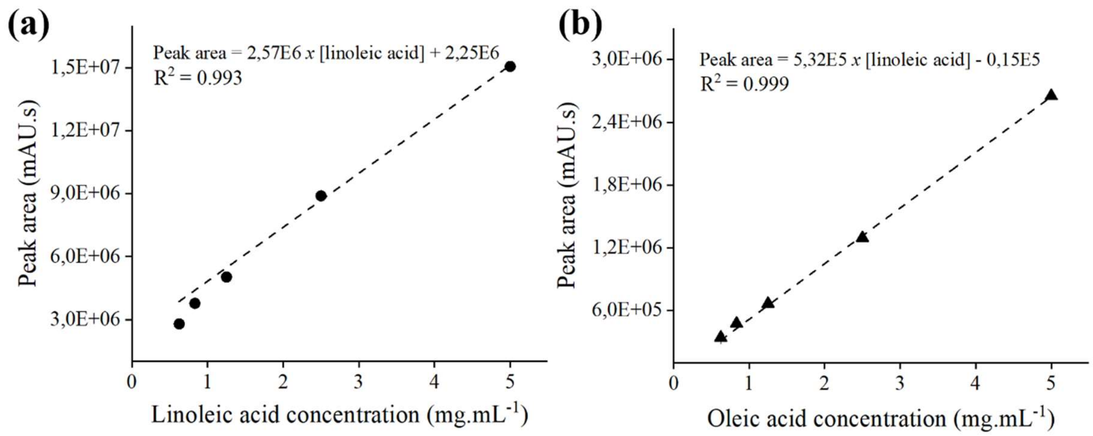

Figure S1. UV response of linoleic and oleic acids at different concentrations.
